# A Case Report of a Female Patient With Hodgkin Lymphoma Localized in the Central Nervous System and With Concomitant Pulmonary Lymphomatoid Granulomatosis

**DOI:** 10.3389/fneur.2020.00963

**Published:** 2020-09-08

**Authors:** Dariusz Szczepanek, Justyna Szumiło, Filip Stoma, Agnieszka Szymczyk, Bożena Jarosz, Aleksandra Szczepanek, Marek Hus, Tomasz Trojanowski, Ewa Wasik-Szczepanek

**Affiliations:** ^1^Chair and Department of Neurosurgery and Paediatric Neurosurgery, Medical University of Lublin, Lublin, Poland; ^2^Chair and Department of Clinical Pathomorphology, Medical University of Lublin, Lublin, Poland; ^3^Chair and Department of Haematooncology and Bone Marrow Transplantation, Medical University of Lublin, Lublin, Poland; ^4^Department of Clinical Transplantology, Medical University of Lublin, Lublin, Poland

**Keywords:** primary Hodgkin lymphoma, lymphoma of the central nervous system, pulmonary lymphomatoid granulomatosis, Epstein-Barr virus, latent membrane protein 1

## Abstract

The involvement of the central nervous system (CNS) in Hodgkin lymphoma (HL) has been rarely reported, especially in its primary isolated form. Herein, we present a case of a 33-year-old woman, who received immunosuppressive treatment due to ulcerative colitis (at the beginning azathioprine and sulfasalazine, changed to mesalazine), with repetitive episodes of loss of consciousness for a few weeks and with no other symptoms. Magnetic resonance imaging scans of the head revealed a tumor in the lateral part of the left temporal lobe and in the cerebellum. Moreover, a subsequent computed tomographic scan of the chest revealed diffuse tumorous lesions in the lungs. The brain tumor was resected and a tumorous lesion resected from the lungs was biopsied. The histopathological analysis confirmed the final diagnosis of HL localized in the CNS with concomitant pulmonary lymphomatoid granulomatosis (LYG) grade 1. After the patient underwent radiotherapy and chemotherapy, the patient showed complete regression of lesions in the CNS and lungs, which was confirmed by positron emission tomographic scan. LYG and CNS-HL are rare proliferative disease derived from lymphocytes B and associated with EBV infections. An association between LYG and other autoimmune disorders has been reported, but to the best of our knowledge, this is the first case of the CNS-HL associated with lymphatoid granulomatosis.

## Introduction

The involvement of the CNS in neoplastic lesions of the lymphatic system is most commonly observed in the case of non-Hodgkin lymphoma (NHL). HL localized in the central nervous system (CNS-HL) has been rarely reported, especially in its primary isolated form (1). It is a specific clinical case to observe the concomitant existence of another rare lymphoproliferative process, such as pulmonary lymphomatoid granulomatosis (LYG). To the best of our knowledge, this is the first case of the CNS-HL associated with lymphatoid granulomatosis.

## Case Presentation

**Figure 1:**
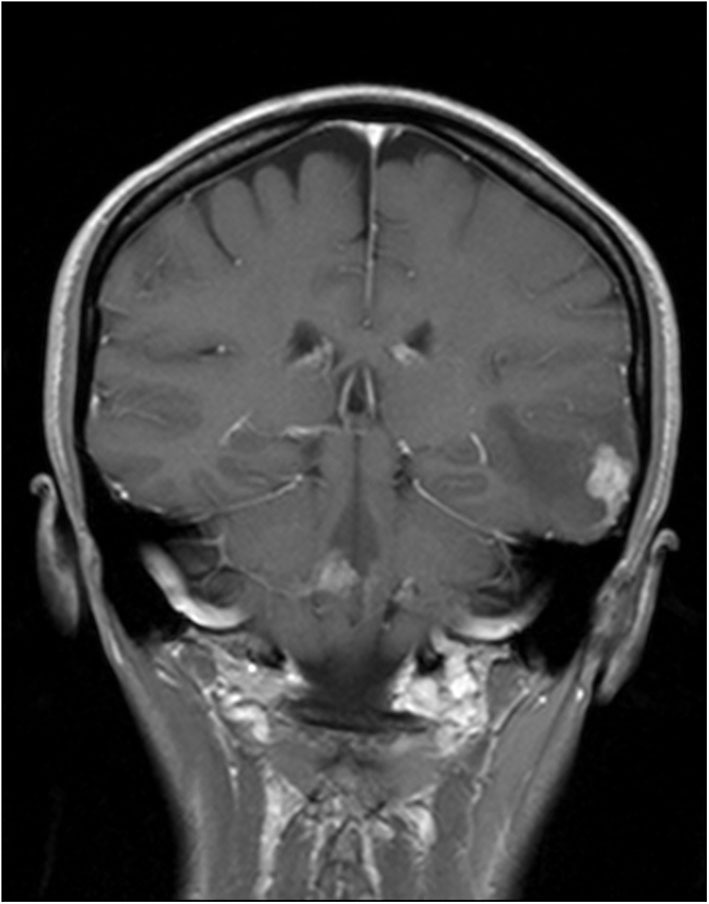
Tumor in the lateral part of the left temporal lobe and the cerebellum in magnetic resonance imaging (MRI) scans.

A 33-year-old female patient was admitted to the Department of Neurosurgery in September 2013 due to repetitive episodes of loss of consciousness for a few weeks. The patient reported no other symptoms. For the past 6 years, she was on immunosuppressive treatment due to ulcerative colitis (at the beginning azathioprine and sulfasalazine (2 years), and next mesalazine). The magnetic resonance imaging (MRI) scans of the head revealed the tumor present in the lateral part of the left temporal lobe and the cerebellum (Figure 1). After the total resection of the tumor, the samples were histopathologically evaluated. At the same time, a computed tomographic (CT) scan revealed diffuse tumorous lesions in the lungs. After resection, a biopsy was performed on one of the lesions.

**Figure 2:**
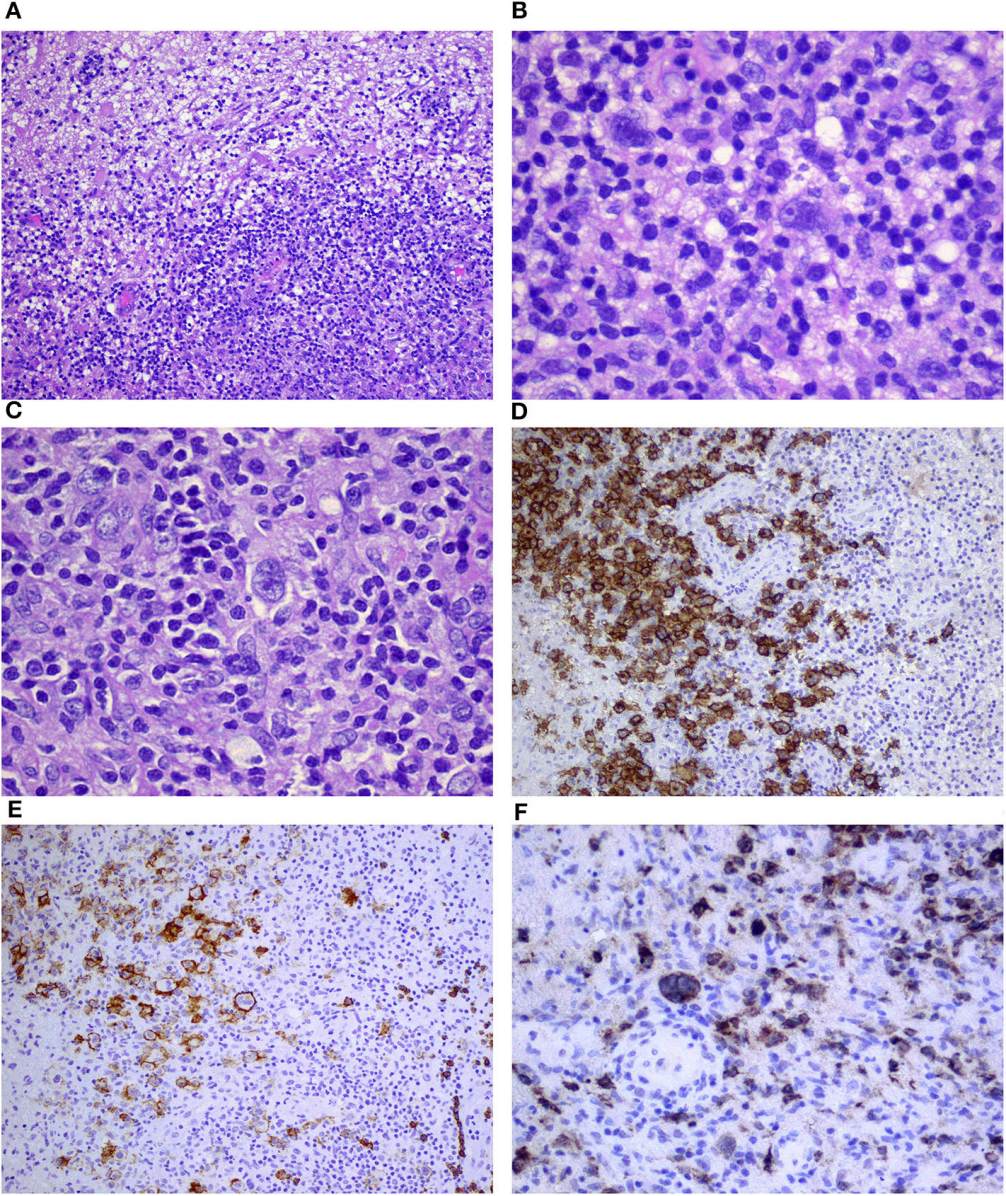
Necrotic area of the lung surrounded by atypical lymphoid cells **(A,B)**; dense lymphocytic infiltration of the wall of the blood vessel **(C)**; and positive immunostaining for CD20 **(D)**, CD30 **(E)**, and EBV/LMP1 **(F)** corresponding to grade 1 lymphomatoid granulomatosis (magnification **A**–10×; **B,D–F**–20×; **C**–40×).

Frozen section of the lesion resected from the right lung was examined (No 18502/13). The specimen was measuring 4.5 × 2.5 × 1.5 cm in size, which showed the presence of whitish-gray nodules. The cross-section of the specimen revealed irregular and slightly brown infiltration. On microscopic examination, the specimen revealed bronchopneumonia with focal necrosis and fibrosis of the lung, which raised the suspicion of lymphoma. However, on further evaluation of the formalin-fixed paraffin-embedded sections, we observed many irregular necrotic areas, as well as fibrinous exudate with many macrophages and a few neutrophils in the alveoli (Figures 2A–C). We also observed sparse giant multinucleated cells, lymphocytic infiltration around the blood vessel, and necrotic areas together with focal fibrosis. In some blood vessels, thrombi including organized ones were also revealed. Additional staining methods, namely, van Gieson, Warthin–Starry, and azan were performed. Necrotizing pneumonia was ultimately diagnosed.

**Figure 3:**
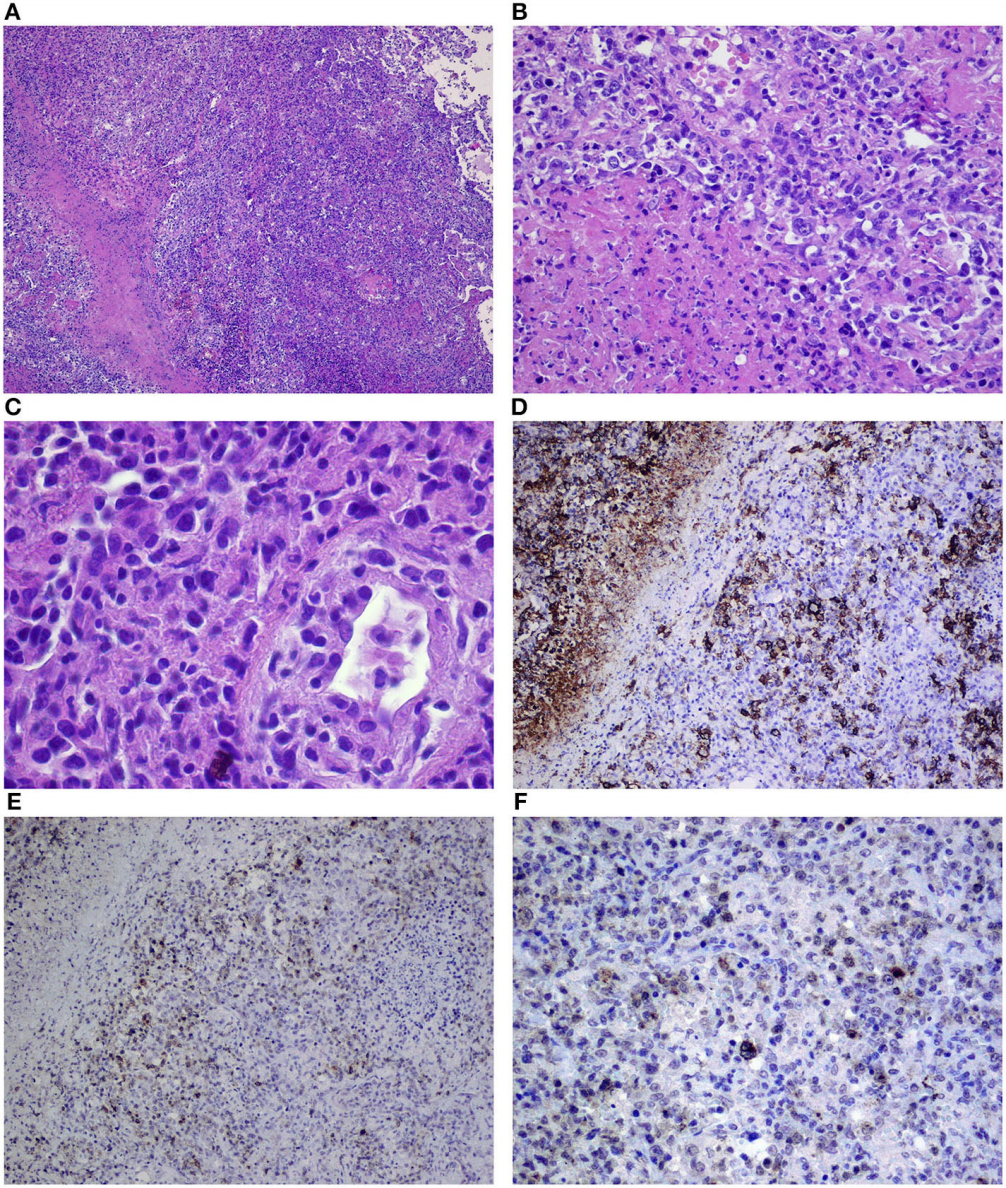
Dense infiltration of the brain composed of Reed–Sternberg cells, small lymphocytes, and macrophages **(A–C)** corresponding to the classical Hodgkin lymphoma of the central nervous system, mixed cellularity (MCcHL), and positive immunostaining for CD30 **(D)**, CD20 **(E)**, and EBV/LMP1 **(F)** in Reed–Sternberg cells (objective magnification **A**–10×; **B,C**–40×; **D–F**–20×).

Samples obtained from the posterior part of the left temporal lobe of the brain (No: 13727) were small grayish and around 0.5–1 cm in size. On microscopic examination, multiple focal points of infiltrations composed mainly of small T cells (CD3+) and macrophages (CD68+) with dispersed giant cells of Reed–Sternberg morphology were observed (Figures 3A–C). The following immunophenotype of the aforementioned cells were revealed: CD45 (+, weaker than in T cells), CD30 (+, strong cytoplasmic and membranous), CD15 (+/–, weak cytoplasmic and in Golgi system in some cells), PAX5 (+, slightly weaker than in B cells), CD20 (+, membranous, weaker than in B cells), OCT (+/–, strong nuclear on the majority of the cells), BOB1 (–/+, trace in single cells), CD10 (–), Bcl6 (–), ALK1 (–), CD3 (–), S100 (–), and CD1a (–). Prognostic markers on Hodgkin and Reed–Sternberg (HRS) cells were as follows: Bcl2 (+, cytoplasmic, weaker than T cells), Ki67 (+, strong in almost all HRS cells), and CD68 (+++) in many concomitant macrophages (category 3) (Figures 3D,E). Classical Hodgkin lymphoma (cHL) of the CNS, mixed cellularity subtype (MCcHL) was diagnosed.

The samples from the brain were consulted and the diagnosis was confirmed. Correlation between the reactivation of Epstein-Barr virus (EBV) with the positive EBV/LMP1 immunostaining was emphasized (Figure 3F). Subsequently, the samples from the lung were reevaluated. A few slightly larger B cells (CD20+, CD30+, CD15–, and EBV/LMP1+) admixed with T cells (CD3+) were found (Figures 2D–F). The final diagnosis was changed to grade 1 LYG with lung involvement. In trephine biopsy (No: 30601/13) lymphomatous infiltration of the bone marrow was not observed.

Evaluation of the cerebrospinal fluid showed no abnormalities. The patient was HIV negative. The following systemic polychemotherapy was introduced: BEACOPP+ DepoCyte® 2 courses), radiotherapy (30 Gy), and CHOP (2 courses). Subsequently, complete regression of lesions in the CNS and lungs was confirmed by a positron emission tomographic (PET) scan.

## Discussion

CNS-HL is estimated in only 0.02% of the patients with the systemic form of the disease (1). It usually takes place either already when stating the diagnosis, or on recurrence or progression of HL (2). The isolated form of CNS-HL is only reported in the literature as single cases (3). One of the most complex reports on all primary CNS-HL cases between 1980 and 2013 is the report published by Kresak et al. (4). The authors showed no correlation between CNS-HL and specific CNS location or neurological symptoms. In six out of seven patients, EBV infection was proven (no such tests were performed in the other nine patients), based on the diagnosis made by histopathological evaluation of the tissue sample and in few cases, by the evaluation of cerebrospinal fluid. Radiotherapy after resection of the lymphoma was the most common treatment modality (12/16 cases) (4).

Complete resection of the lymphoma was performed and subsequently cytostatic treatment and radiotherapy were administered to the patient. It is noteworthy that complete resection of the lymphoma as a therapeutic modality for primary NHL is controversial and has been rarely performed. The most commonly performed neurosurgical procedure is limited to stereotactic biopsy as a part of the diagnostic process. One of the many arguments that question the relevance of radical surgical procedure is the conclusion of post mortem examinations. These examinations proved leukemic infiltration even in normal brain tissue (whole-brain disease) (5).

However, so far, there is no data related to the involvement of CNS in HL. The radiological picture of CNS-HL is commonly uncharacteristic. It may sometimes resemble ischemic or inflammatory lesions or even other tumors (i.e., meningioma, glioblastomas, or metastases) (6, 7). CNS involvement in HL tends to have less mass effect and edema. In addition, necrosis, hemorrhage, and calcification are rare in these cases (7). In differentiating other changes in the brain, you can consider expanding diagnostics with single-photon emission computed tomography (SPECT), MR spectroscopy (MRS), or perfusion MRI (8).

The mechanism that leads to the involvement of CNS in HL is unknown. In the case of coexistence of a systemic disease, a direct transition of neoplastic process from surrounding skull bones or hematogenous dissemination has been suggested (9). However, large-sized Reed–Sternberg cells effectively restrict their transition into the perivascular spaces of the CNS, which prevents the formation of CNS-HL (10).

According to the literature, the isolated form of CNS-HL is associated with a better prognosis than that of CNS-HL in the phase of recurrence or HL progression (4). LYG is a rare proliferative disease derived from lymphocytes B and is associated with EBV infections (11). It has been most commonly described for the lungs (about 90% of all the cases), followed by the skin (25–50%) and CNS (25–35%). The involvement of lymph nodes, spleen, or bone marrow has been described in the advanced stages of the disease (12).

An association between LYG and other autoimmune disorders (e.g., Sjogren's syndrome, rheumatoid arthritis, sarcoidosis, and ulcerative colitis), as well as congenital or acquired immunodeficiencies, has been reported. Patients with pulmonary form of LYG usually complain of numerous symptoms (shortness of breath, cough, and chest pain); however, sometimes the diagnosis may be stated when evaluating the lungs due to other reasons (like in our patient). The radiological picture usually shows numerous nodules of different sizes in both lungs (13).

LYG is an angiocentric and angiodestructive process with the presence of large B cells (EBV+) in the infiltrated tissues. They show the expression of CD20, variably CD30, and no expression of CD15, which leads us to exclude HL. Numerous reactive T cells are characteristic in LYG (13). In addition, LYG is characterized by foci of necrosis associated with angiogenic ischemic processes. It is thought to be caused by the direct infiltration of blood vessels by T cells. Chemokines IP-10 and Mig induced by EBV have also been suggested to play a role in the aforementioned process (14). The clinical course of LYG is varied and the method of treatment mainly depends on the stage that is based on the number of atypical EBV+ B cells. Some patients show spontaneous remission or no progression for a long time. However, in the case of an advanced stage of LYG, chemotherapy is required (e.g., CHOP, ICE, or hyperCVAD) in combination with the administration of monoclonal antibodies, anti-CD20 (13).

In this study, cells with the expression of latent membrane protein 1 (LMP-1; viral protein responsible for oncogenesis) were detected in the tissue samples obtained from the CNS lesion and lungs of the patient. This proves a significant role of EBV in the development of CNS-HL and pulmonary LYG in the presented patient.

LMP-1 is an integral cell membrane protein whose role is to protect infected lymphocytes from apoptosis through the increased expression of some anti-apoptotic genes, such as Bcl-2, Mcl-1, and A20. However, we still do not know why two separate lymphoproliferative processes, triggered most probably by the same factor, developed at the same time in the same patient. The influence of EBV on the expression of receptor proteins, signaling, maturation, and differentiation of the infected cell present only a part of the issues, which requires further investigation (14).

The lungs, liver, and bone marrow are among the non-lymphatic organs most commonly affected by HL (15). In the case of diagnosis of HL based on histopathological examination of the lymph node, sometimes changes in the lung are not usually verified (they are thought to be related to HL). It is worth to consider the legitimacy of histopathological assessment in the absence of response to standard HL chemotherapy. It may turn out that the coincidence of HL and LYG occurs more often than it may seem, which would thus allow an in-depth explanation of the etiopathogenesis of the simultaneous occurrence of both conditions. It is very difficult to interpret considering that one case has been described so far (16–18). It is significant, that the MC subtype of HL is the most associated with EBV infection (19) (such as was present in our patient). There is also indicated impact of immune disorders (e.g., prior immunosuppression) on the development of both disease processes (20).

The diagnostic process for both the CNS and lung in the patient's work required detailed and complicated diagnostics. In the lungs, the possibility of tuberculosis or other inflammatory processes that excluded intensive chemotherapy could be considered. The simultaneous occurrence of HL and tuberculosis has already been described in the literature (16–18). Jehanno et al. addressed the diagnostic problems of possible coincidence of both conditions using the PET/CT imaging technique (21). These data indicate the important, clinically important importance of the description of this case.

## Ethics Statement

This study was approved by the Ethics Committee of the Medical University of Lublin (KE No. 0254/159/2014). Study was performed in accordance with the ethical standards of the Declaration of Helsinki. There was no additional invasive test or experimental drugs used out of order for the patient. Written informed consent was obtained from the patient for participation and publication of this case report.

## Author Contributions

DS, EW-S, JS, FS, ASzy, BJ, ASzc, MH, and TT was responsible for the data collection and analysis, interpreted the results, and contributed to manuscript writing. TT, DS, and EW-S contributed to the interpretation of the data and critical revision of the manuscript for important intellectual content. DS and EW-S designed the study. All authors contributed to the article and approved the submitted version.

## Conflict of Interest

The authors declare that the research was conducted in the absence of any commercial or financial relationships that could be construed as a potential conflict of interest.
